# P2Y2 promotes fibroblasts activation and skeletal muscle fibrosis through AKT, ERK, and PKC

**DOI:** 10.1186/s12891-021-04569-y

**Published:** 2021-08-11

**Authors:** Mengjie Chen, Haibing Chen, Yonggui Gu, Peng Sun, Jianxiong Sun, Haojun Yu, Hongliang Zheng, Donghui Chen

**Affiliations:** 1 Department of Otorhinolaryngology, Changhai Hospital of Navy Medical University, 168 Changhai Road, Shanghai, 200433 China; 2 Department of Otorhinolaryngology, The First Affiliate Hospital of Nanjing Medical University, 300 Guangzhou Road, Nanjing, 210029 Jiangsu China; 3 Department of Otorhinolaryngology, Jingjiang People’s Hospital, Jingjiang, 214500 Jiangsu China; 4 Department of Otorhinolaryngology, The First Affiliate Hospital of Soochow University, Suzhou, 215006 Jiangsu China

**Keywords:** P2Y2, Muscle atrophy, Muscular fibrosis, Fibroblast, Extracellular matrix

## Abstract

**Background:**

Skeletal muscle atrophy and fibrosis are pathological conditions that contribute to morbidity in numerous conditions including aging, cachexia, and denervation. Muscle atrophy is characterized as reduction of muscle fiber size and loss of muscle mass while muscle fibrosis is due to fibroblasts activation and excessive production of extracellular matrix. Purinergic receptor P2Y2 has been implicated in fibrosis. This study aims to elucidate the roles of P2Y2 in sleketal muscle atrophy and fibrosis.

**Methods:**

Primary muscle fibroblasts were isolated from wild type and P2Y2 knockout (KO) mice and their proliferating and migrating abilities were assessed by CCK-8 and Transwell migration assays respectively. Fibroblasts were activated with TGF-β1 and assessed by western blot of myofibroblast markers including α-SMA, CTGF, and collagen I. Muscle atrophy and fibrosis were induced by transection of distal sciatic nerve and assessed using Masson staining.

**Results:**

P2Y2 KO fibroblasts proliferated and migrated significantly slower than WT fibroblasts with or without TGF-β1.The proliferation and ECM production were enhanced by P2Y2 agonist PSB-1114 and inhibited by antagonist AR-C118925. TGF-β1 induced fibrotic activation was abolished by P2Y2 ablation and inhibited by AKT, ERK, and PKC inhibitors. Ablation of P2Y2 reduced denervation induced muscle atrophy and fibrosis.

**Conclusions:**

P2Y2 is a promoter of skeletal muscle atrophy and activation of fibroblasts after muscle injury, which signaling through AKT, ERK and PKC. P2Y2 could be a potential intervention target after muscle injury.

**Supplementary Information:**

The online version contains supplementary material available at 10.1186/s12891-021-04569-y.

## Background

Muscle atrophy may occurs as a systemic response to fasting and various diseases including cancer, AIDS, cardiac and renal failure, and sepsis or in specific muscles due to denervation or inactivity [1–3]. The degradation of both myofibrillar and soluble proteins is accelerated during atrophy. Denervation causes several biochemical, morphological and physiological changes in the muscle fiber, which might proceed to terminal atrophy [4]. Although muscle atrophy is a result of inhibition of protein synthesis and / or acceleration of proteasomal degradation, many singaling pathways have been implicated in skeletal muscle atrophy [5].

Fibroblasts are metabolically active cells and exist in most tissues of the body, which play critical roles maintaining tissue integrity, regulating extracellular matrices, interstitial fluid volume and pressure, and wound healing [6, 7]. Fibroblasts are characterized as vimentin^+^, α-smooth muscle actin (α-SMA)^−^, and desmin^−^, whose ability to produce and respond to growth factors maintains the homeostasis of adjacent cell types such as epithelial and endothelial cells [6]. Fibroblasts are the dominant type of cells within the connective-tissue cell family and its physiological roles include producing ECM production and regulating tissue homeostasis and inflammation and the differentiation of the surrounding cells [8]. Moreover, fibroblasts are also the source of the matrix metalloproteinases (MMPs) [9] that are involved in matrix remodeling, cell motility, proliferation, and death. The array of growth factors secreted by fibroblasts further facilitate interactions among surrounding cells [10]. In skeletal muscle, fibroblasts produce the amority of the extracellular matrix (ECM) [11, 12] even though they only account for a small number of cells [13]. These fibroblasts reside in the interstitial space between muscle fibers and play a critical role in maintaining muscle structure. Moreover, fibroblasts are required for muscle regeneration as the differentiation of satellite cells is premature and muscle fibers are poorly regenerated with decreased diameters when fibroblasts are ablated [14].

Activated fibroblasts are the main effectors for the initiation of fibrosis as a result of excessive collagen deposition and an improper extracellular matrix [15]. Fibrobalasts are activated by TGF-β1 and other factors and differentiate into myofibroblasts, which are contractile and express α-SMA and the synthesize extracellular matrix proteins. Both acute (e.g. stroke or muscle trauma) and chronic (e.g. muscular dystrophy and cerebral palsy) injuries could cause skeletal muscle fibrosis [16, 17]. Regeneration is initiated by muscles after damages but dystrophin is lack from the regenerated muscle, which eventualy causes myofiber necrosis. The muscle damage induces an inflammatory response cascade involving the inflitration of inflammatory cells into the injured tissue [18, 19]. These inflammatory cells release soluble mediators (alarmins, cytokines, chemokines) that promote the activation of fibroblasts and ECM production. Moreover, muscular dystrophies induced chronic inflammation results in a positive feedback secretion of inflammatory cytokines from fibroblasts and additional ECM production [18–20]. The chronic inflammation induces persistent activation of skeletal muscle fibroblasts and ECM overproduction in the diseased muscle, which ultimately causes muscle fibrosis.

P2Y receptors are a family of purinergic G protein-coupled receptors which are stimulated by nucleotides. There are 8 mammanlian P2Y family members identified so far: P2Y1, P2Y2, P2Y4, P2Y6, P2Y11, P2Y12, P2Y13 and P2Y14 [21]. P2Y2 is strongly activated by both ATP and UTP with approximately equal potency while it is insensitive to or only weakly activated by ADP or 2-methylthio ADP (2MeSADP) [22]. P2Y2 is coupled to G_q/o/,12_ and signals through phospholipase C (PLC) and Ca^2+^ to induce cell proliferation and migration [23]. Deletion of P2Y2 gene completely blocked ATP and UTP induced Ca^2+^ responses in fibroblasts [24] and impaired skin wound healing [25]. Moreover, P2Y2 is overexpressed in idiopathic pulmonary fibrosis patients or the lungs of bleomycin treated mouse [26]. P2Y2 has been shown to play important roles in in human airway epithelia repair and remodeling through regulating the proliferation and migration of pulmonary endothelial cells, smooth muscle cells and fibroblasts, collagen deposition, and neovascularization [27]. UTP activates P2Y2 receptor in cardiac fibroblast to elicit a profibrotic response [28]. However, it is not clear whether P2Y2 plays a role in denervation caused muscle atrophy and fibrosis. This study aims to investigate the role of P2Y2 receptor in the activation of myofibroblasts and skeletal muscle fibrosis.

## Material and methods

### Ethics approval and consent to participate

The animal protocol was approved by the institutional animal care and usage committee of Navy Medical University (Approval #: NMU-20190037). All procedures were performed strictly conforming with the approved protocol and the Guidelines for Laboratory Animal Wellness issued by the Ministry of Science and Technology of China, and the study was carried out in compliance with the ARRIVE guidelines.

### Skeletal muscle injury model

C57B/J and P2Y2 knockout mice were purchased from Shanghai Nanfang Model Animal Biotechnology Co (Shanghai, China). After quarantine and acclimation, 8 week old male WT and P2Y2 KO mice were used to establish skeletal muscle injury model. After anesthetizing the mice by intraperitoneal injection of 100 mg/kg ketamine (catalog no. 511485, Merck & CO., Kenilworth, NJ), the skin of the left hind leg was incised and the muscles were separated. The sciatic nerve was exposed and about 1/3 to 1/2 of the distal sciatic nerve was transected and ligated with a size 7 nylon suture. The skin was sutured back and penicillin ointment was applied to the wound area for 3 days and the mice were kept in the SPF facility. Five mice (WT and P2Y2 KO each) were sacrificed by CO_2_ inhalation followed by cervical dislocation and gastrocnemius muscle tissues were removed from the left (injured side) and right (normal control) hind legs of each mouse at 2, 4, and 6 weeks post-injury.

### Masson staining

Muscle fibrosis was evaluated by Masson staining, which was performed using a commercially available kit (ServiceBio, Shanghai, China). The sections were deparaffinized and rehydrated in xylene for 20 min twice, anhydrous ethanol for 5 min twice, 75% ethanol for 5 min, and rinsed with distilled water. The slides were immesed in potassium dichromate working solution overnight and washed with distilled water, immersed in iron hematoxylin staining solution for 3 min and washed in distilled water, immersed in ponceau acid fuchsin for 5–10 min, rinse with distilled water, dipped in molybdophosphoric acid solution for 1–3 min and then in the aniline blue dyeing solution for 3–6 min, differentiated with 1% glacial acetic acid and dehydrated in two rounds of absolute ethanol. The slides were passed through a third round of 100% ethanol for 5 min and then in xylene for 5 min before sealed with neutral gum.

### Isolation of primary fibroblasts from mouse skeletal muscle

Mouse skeletal muscle fibroblasts were isolated using differential attachment method. Briefly, after the wild type and P2Y2 KO mice were sacrificed, the skin of the thighs was carefully removed and the entire hind legs were dissected and placed in a petri dish containing RP-1640. Under a dissecting microscope, the muscles from mouse thighs were dissected out and the blood vessels and bones were removed from the muscles. The muscle tissues were placed in a new petri dish (on ice) and cut into 2 mm^3^ pieces (slurry). The tissue slurry was transferred into a 50 ml centrifuge tube containing 3 ml DPBS. Then dissociation enzyme solution (Collagenase Type 2 final concentration 0.2% and Neutral Protease final conc. 0.5 U/ml) was added, mixed with gentle shake, incubated at 37 °C for 45 min with periodic gentle shake. At the end of incubation, 5 ml fibroblast growth medium (FGM, Millipore-Sigma, Shanghai, China) was added into the tube and gently blew the mixture 5–8 times with a 10 ml pipette. and then add 12 ml of FGM culture solution. The cell suspension was passed through a 70 μm cell strainer and then centrifuged at 300 g at 4 °C for 5 min. The cells were resuspended in 5 ml FGM and passed through a 40 μm disposable strainer. The cells were cultured at 37 °C in a 5% CO_2_ incubator for 40 min. After gentle shaking, The non-adherent cells and culture medium were aspirated and remaining cells were cultured in fresh FGM at 37 °C in a 5% CO_2_ incubator.

### Quantitative real-time polymerase chain reaction (RT-qPCR)

The change of gene expression at mRNA level was assessed with RT-qPCR. RNA was extracted using RNeasy mini kit (Qiagen, Germantown, MD) following manufacturer’s manual. Total RNA (0.5 μg) was reverse-transcribed into cDNA using Invitrogen SuperScript III Reverse Transcriptase kit (ThermoFisher, Shanghai, China). Real-time RT-PCR amplication was carried on an ABI 7500 Fast (Applied Biosystems, Foster City, CA) using SYBR Premix Ex Taq™ kit (Takara, Dalian, China). The PCR primers were ACCCTCAACGCCATCAACAT and CGTCTTGAGTCGTCACTGCT for P2Y2; CTTCGTGACTACTGCCGAGC and AGGTGGTTTCGTGGATGCC for α-SMA; CGATGGATTCCCGTTCGAGT and CGATCTCGTTGGATCCCTGG for Col1a1; TCCGGACACCTAAAATCGCC and TTCATGATCTCGCCATCGGG for CTGF and CCGAGAATGGGAAGCTTGTC and AAGCACCAACGAGAGGAGAA for glyceraldehyde 3-phosphate dehydrogenase (GAPDH). The relative gene expression level was calculated with 2^-ΔΔCt^ method using GAPDH as internal control.

### Western blot

Western blot was performed following previously published method [29] to analyze the changes of specified proteins. Briefly, primary skeletal muscle fibroblasts were lyzed with RIPA buffer (Beyotime Bio, Shanghai, China) supplemented with proteinease inhibitor and phosphatase cocktail (Sigma, St. Louis, MO) and total proteins (40 μg) were resolved on 8% SDS-PAGE gels and transferred onto PVDF membranes. The membranes were blocked in 5% nonfat milk in TBST (50 mM Tris, pH 7.5; 150 mM NaCl; 0.1% Tween 20) for 45 min, incubated with primary antibodies at 4 °C overnight, washed and incubated with proper horseradish peroxidase conjugated secondary antibodies (Jackson ImmunoResearch, West Grove, PA) at room temperature for 60 min before visualized with enhanced chemiluminescence (ECL) reagents (Pierce, Rockford, IL). The primary antibodies used were Col I antibody, a-SMA antibody, CTGF antibody, TGF-β1 antibody, Fibronectin antibody, p-AKT antibody, AKT antibody, p-ERK antibody, PKC antibody, p-PKC antibody and β-Actin Antibody (sc-47,778) were purchased from Santa Cruz Biotech (Shanghai, China).

### Cell counting kit 8 (CCK-8) assay

CCK-8 assay was used to assess the viability and proliferation of skektal muscle fibroblasts. Five thousand fibrolasts per well were seeded in 96 well plates and cultured at 37 °C with 5% CO_2_ for 24 h with or without treatement. Ten microliter of CCK-8 working solution (Beyotime, Shanghai, China) was added to each well and incubated at 37 °C for 2 h before the absorbance was measured at 450 nm using a microplate reader (Molecular Devices, San Jose,CA).

### Migration assay

The role of P2Y2 in primary muscle fibroblast migration was assessed by scratch wound healing assay [30]. Briefly, an aliquot of 200 μl of the fibroblasts (5 × 10^4^/ml) cell suspension (without FBS) was placed into the upper chamber of a Corning Transwell 24 Permeable Support Culture Plate (Sigma, St Lois, MO.) and 500 μl complete medium into the lower chamber. The cells were cultured 12 h before the membrane was fixed with 10% cold methanol at 4 °C for 10 min. The cells on the top side of the membrane were wiped off and the membrane was stained with crystal violet. The number of migrated cells was counted in 5 random fields (× 200).

### Statistical analysis

The data were expressed as mean ± standard deviation. The difference between the groups was determined using one-way analysis of variance (ANOVA) or two-tailed Student’s t-test. A *p* values less than 0.05 was considered statistically significant.

## Results

### Ablation of P2Y2 alleviated denervation induced skeletal muscle atrophy and fibrosis

**Fig. 1:**
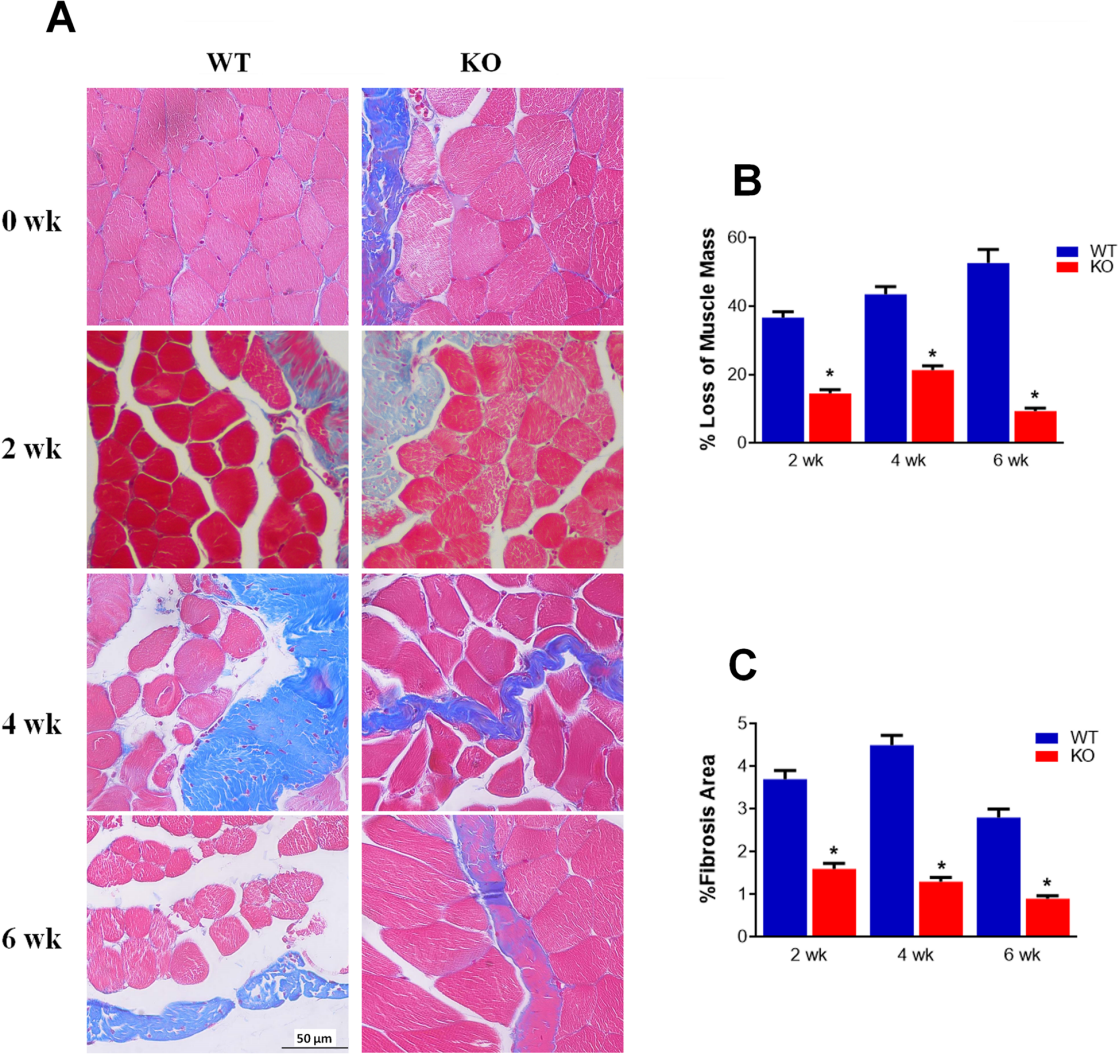
P2Y2 exacerbated denervation caused skeletal muscle atrophy and fibrosis. **a** Representative pictures of masson staining of mouse gastrocnemius muscle different time after denervation. **b** Loss of muscle mass as a percentage of non-denervated control leg mass gastrocnemius at specified time (*n* = 5). **c** Fibrosis area of denervated gastrocnemius muscle was measured with Image J. WT, C57Bl/6 N wild type; KO, P2Y2 knockout. * *p* < 0.05 compared to wild type of the same time point

To investigate the roles of P2Y2 in skeletal muscle injury, we assessed gastrocnemius muscle atrophy and fibrosis of both wild type and P2Y2 KO mice after denervation. The loss of muscle mass in WT mice was deteriorated along the time after denervation (Fig. 1a-b). The muscle mass loss was significantly reduced in P2Y2 KO mice (Fig. 1a-b). Moreover, the loss of muscle mass was continued in wild type mice but stoped in P2Y2 KO mice at 6 weeks after denervation (Fig. 1a-b). At the same time, the interstitial collagens were significantly less in P2Y2 KO mouse gastrocnemius muscle than that of wild type mice at all time points after denervation (Fig. 1a and c).

### P2Y2 enhances the proliferation of skeletal muscle fibroblasts

**Fig. 2:**
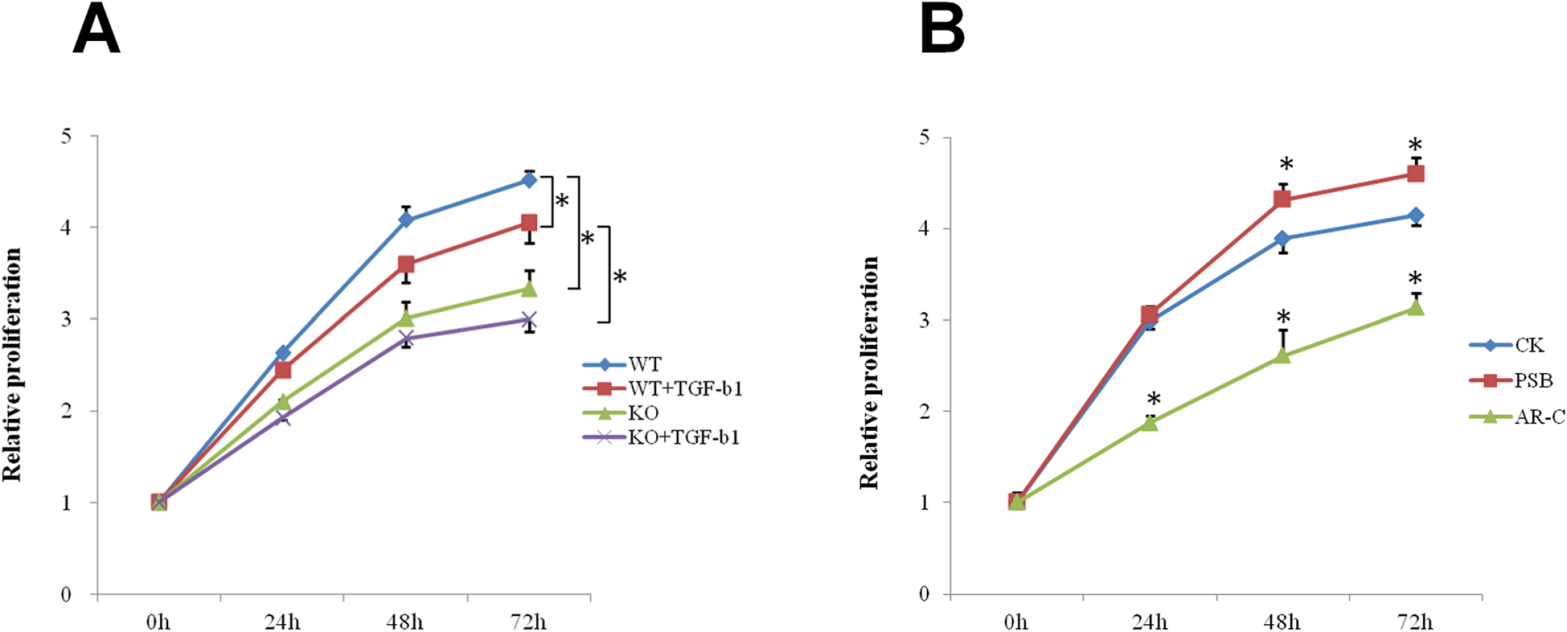
P2Y2 promoted the proliferation of skeletal muscle fibroblasts. **a** Primary fibroblasts isolated from wild type or P2Y2 KO mice were cultured at 37 °C with or without TGF-β1 and assayed with cell counting kit-8 at 0, 1, 2 and 3 days. **b** Wild type skeletal muscle fibroblasts were treated with 10 μM PSB-1114 or 100 μM AR-C118925 for 6, 12, 24, 48, 64, and 72 h and assayed with cell counting kit-8. WT, C57Bl/6 N wild type; KO, P2Y2 knockout; PSB, PSB-1114; AR-C, AR-C118925. * *p* < 0.05 between specified two groups (**a**) or compared to wild type (**b**)

As P2Y2 knockout alleviated denervation-caused muscle loss and fibrosis, we next probed the roles of P2Y2 in regulating the biological behavior and function of muscle fibroblasts. Skeletal muscle fibroblasts (Fig. S1) from P2Y2 knockout mice proliferated significantly slower than those from wild type mice (Fig. 2a). TGF-β1 treatment inhibited the proliferation of both wild type and P2Y2 KO fibroblasts but P2Y2 KO fibroblasts still proliferated much slower than wild type fibroblasts (Fig. 2a). P2Y2 antagonist AR-C118925 significantly inhibited while agonist PSB-1114 slightly enhanced the proliferation of WT fibroblasts (Fig. 2b).

### P2Y2 promotes the motility of skeletal muscle fibroblasts

**Fig. 3:**
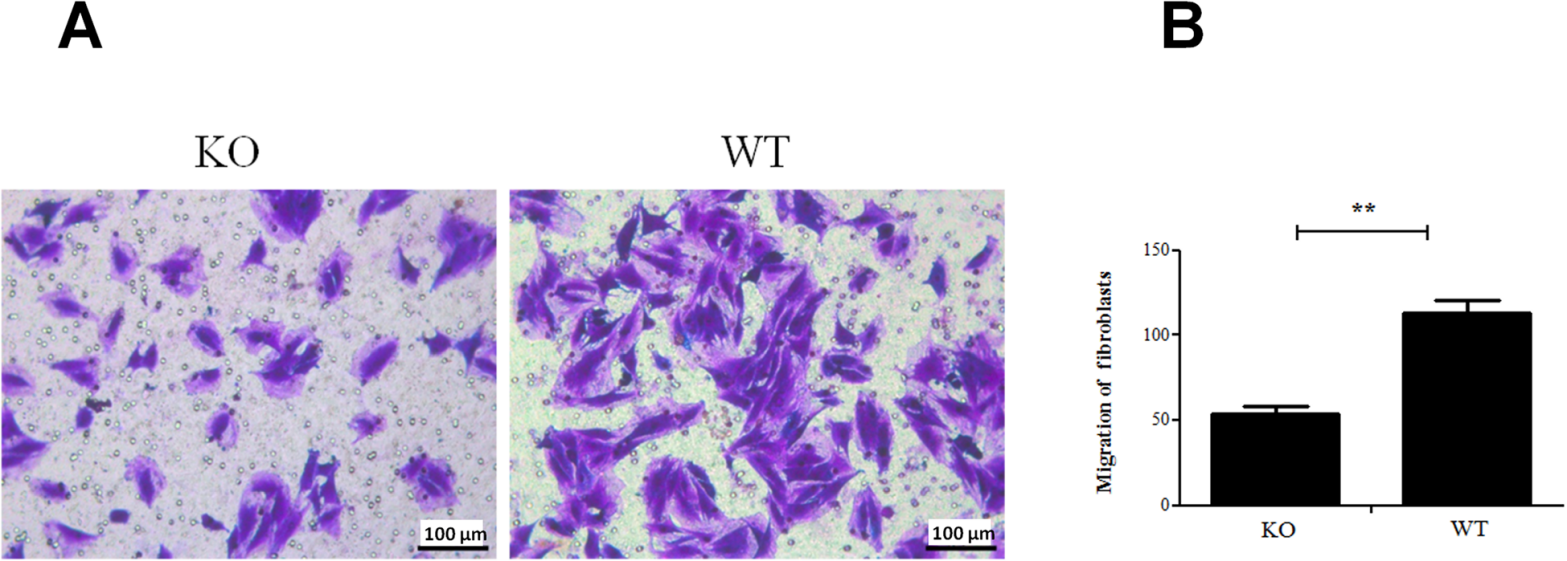
Cell migration was reduced by P2Y2 ablation. **a** Wild type and P2Y2 knockout mouse skeletal muscle fibroblasts were subjected to Transwell migration assay and typical pictures were shown. **b** quantitative analysis of migration assay results. WT, C57Bl/6 N wild type; KO, P2Y2 knockout. **, *p* < 0.01

Next the role of P2Y2 in cell motility was assessed by Transwell migration assay. The migration rate of P2Y2 KO fibroblasts was about 2-time lower than that of WT fibroblasts (Fig. 3).

### Fibroblast activation and ECM production were promoted by P2Y2

**Fig. 4:**
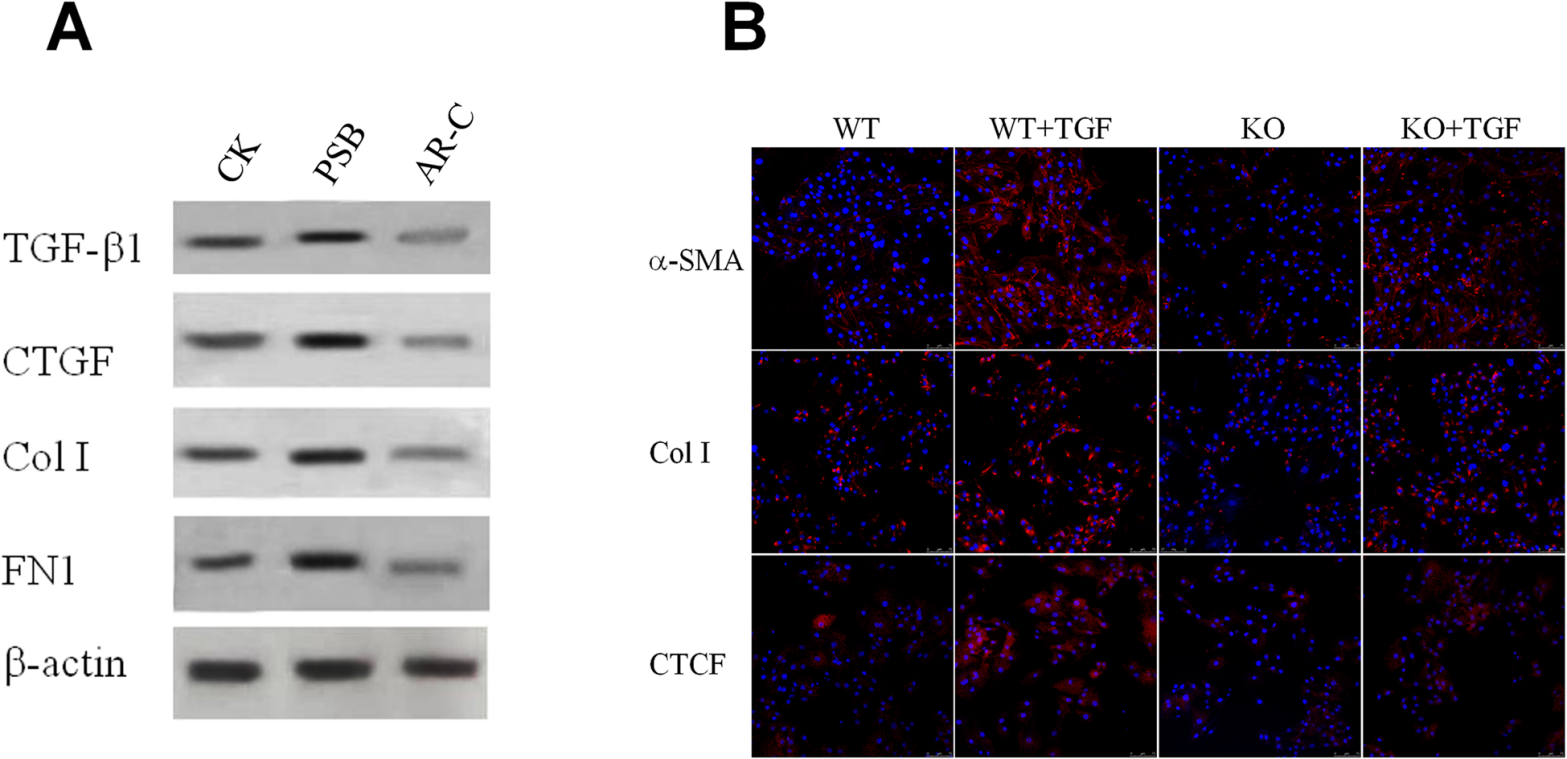
P2Y2 enhanced fibrotic activity of skeletal muscle fibroblasts. **a** Western blot detection of the expression of fibrotic genes TGF-β1, CTGF, Collagen 1, and Fibronectin 1 after mouse skeletal muscle fibroblasts were treated with 10 μM PSB-1114 or 100 μM AR-C118925 for 12 h. B. Immunofuorescence detection of α-SMA, collagen I, and CTCF in wild type and P2Y2 KO mouse skeletal muscle fibroblasts treated with or without 10 μM TGF-β1. WT, C57Bl/6 N wild type; KO, P2Y2 knockout; PSB, PSB-1114; AR-C, AR-C118925; TGF, TGF-β1

The protein levels of TGF-β1, CTGF, Collagen I, and fibronectin of WT fibroblasts were substantially increased by P2Y2 agonist PSB-1114 and inhibited by P2Y2 antagonist AR-C118925 (Fig. 4a). Moreover, P2Y2 null fibroblasts were drastically insensitive to exogenous TGF-β1 induced activation. The upregulation of myofibroblast marker α-SMA, Col I and CTGF induced by TGF-β1 was very much subdued in P2Y2 null fibroblasts (Fig. 4b).

### P2Y2 signaling through AKT, ERK, and PKC

**Fig. 5:**
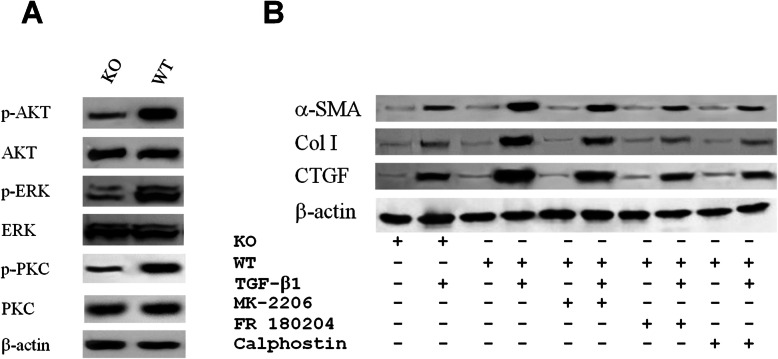
AKT, ERK and PKC mediated the fibrotic activity of P2Y2. **a** Western blot detected the activation of AKT, ERK, and PKC in wild type and P2Y2 KO mouse skeletal muscle fibroblasts. **b** The protein levels of α-SMA, collagen I, and CTGF in mouse skeletal muscle fibroblasts treated with TGF-β1 in the presence or absence of MK-2206 2HCl, FR 180204, and Calphostin C were detected by western blot. WT, C57Bl/6 N wild type; KO, P2Y2 knockout

To identify the downstream signaling pathways of P2Y2 receptor, we compared the activation of different protein kinases by P2Y2 agonist in WT and P2Y2 null fibroblasts. Deletion of P2Y2 drastically reduced the phosphorylation of AKT, ERK, and PKC (Fig. 5a). Moreover, the expression of myofibroblast markers α-SMA, Col I and CTGF in WT fibroblasts was considerably inhibited by AKT inhibitor MK-2206 2HCl, ERK inhibitor FR 180204, and PKC inhibitor Calphostin C (Fig. 5b).

### P2Y2 promotes skeletal muscle fibroblast proliferation and migration through AKT, ERK, and PKC

**Fig. 6:**
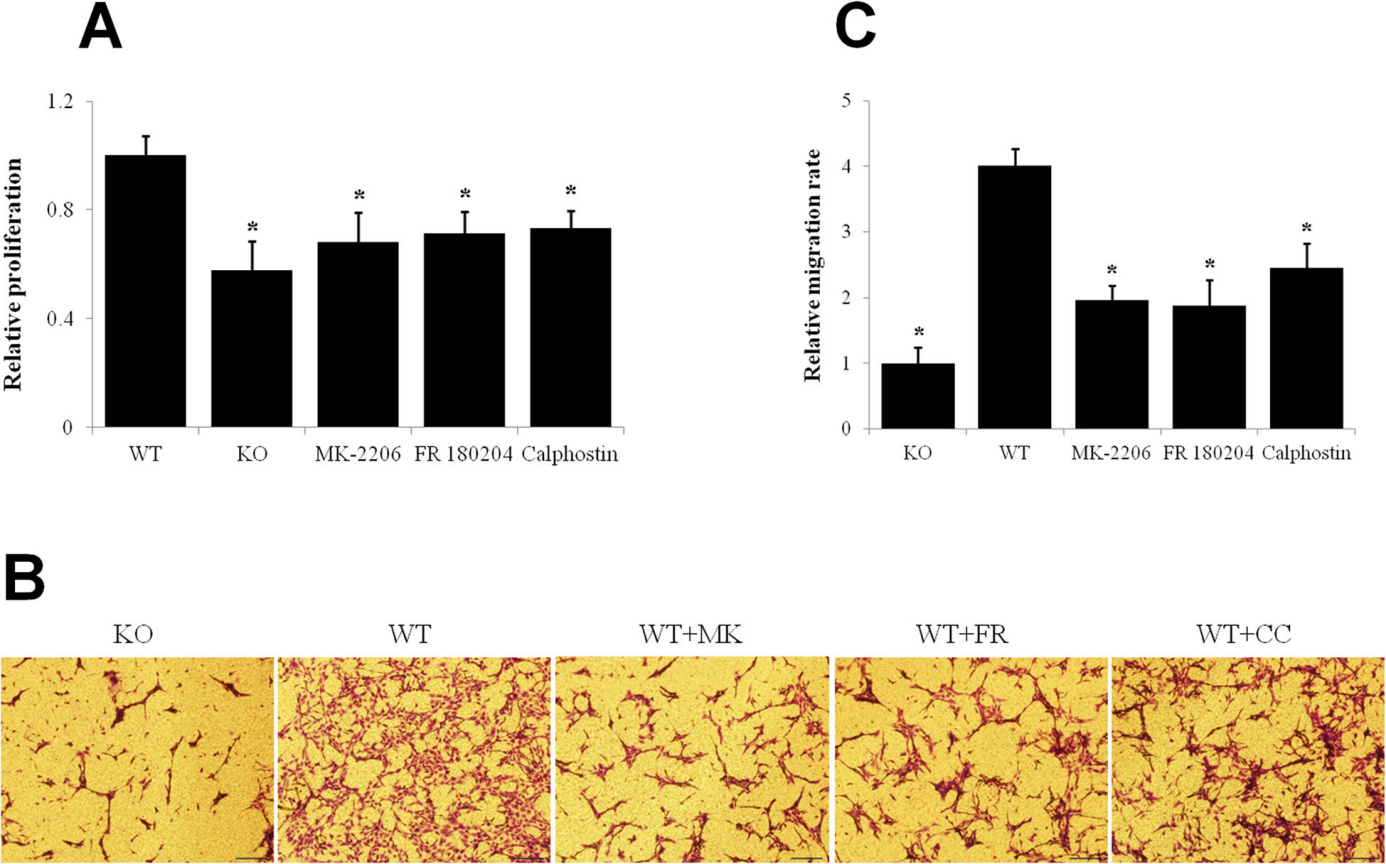
P2Y2 promotes skeletal muscle fibroblast proliferation and migration through AKT, ERK, and PKC. **a** Mouse skeletal muscle fibroblasts were treated with MK-2206 2HCl, FR 180204, and Calphostin C for 72 h and assessed by CCK-8 assay. **b** Mouse skeletal muscle fibroblasts were treated with MK-2206 2HCl, FR 180204, and Calphostin C and subjected to Transwell migration assay. **c** Quantitative analysis of migration rate * *p* < 0.05 compared to WT. WT, C57Bl/6 N wild type; KO, P2Y2 knockout; MK, MK-2206 2HCl; FR, FR 180204; CC, Calphostin C. * *p* < 0.05 compared to WT

The proliferation rate of WT fibroblasts was much higher than P2Y2 knockout fibroblasts and the proliferation of WT fibroblasts was significantly inhibited by AKT inhibitor MK-2206 2HCl, ERK inhibitor FR 180204, and PKC inhibitor Calphostin C (Fig. 6a). Meanwhile, inhibition of AKT, ERK, and PKC also suppressed WT fibroblast migration, which was markedly higher than P2Y2 knockout fibroblast (Fig. 6b-c).

## Discussion

The current study elucidated the role of P2Y2 in the proliferation, migration, and activation of skeletal muscle fibroblasts. Knockout P2Y2 inhibited the proliferation and migration of fibroblasts at both basal and TGF-β1 treated levels. Activation of P2Y2 increased and antagnization of P2Y2 reduced the proliferation of fibroblasts. The activation of AKT, ERK and PKC in fibroblasts by ATP in P2Y2 null fobroblasts was significantly weaker than in WT fibroblasts. Inhibiting AKT, ERK and PKC blocked the effects of P2Y2 in fibroblast proliferation, migration and activation.

Besides promoting skeletal muscle fibrosis and muscle mass loss after injury, P2Y2 receptor has been shown to regulate the activation and function of fibroblasts under various conditions and diseases. Extracellular ATP could more strongly enhance IL-6 production in systemic sclerosis fibroblasts than in normal fibroblasts, which was significantly inhibited by selective P2Y2 receptor antagonists AR-C118925XX. Moreover, AR-C118925XX also inhibited ATP-induced phosphorylation of p38 and collagen I production in systemic sclerosis fibroblasts [31]. ATP induced transient increases in intracellular Ca^2+^ concentration and contraction of rat intestinal subepithelial myofibroblasts were inhibited by selective P2Y2 antagonist AR-C118925 [32]. Knockout P2Y2 blunted UTP-induced Ca^2+^ responses and inhibited ERK and PKC activation in mouse cardia fibroblasts, leading to moderation of fibrotic remodeling and fibrosis of hearts [33]. In a full-thickness skin wound model, WT mice had significantly decreased wound size than P2Y2 knockout mice 14 days after injury, and silencing P2Y2 expression in mouse skin fibroblasts reduced their migration and ECM production [25]. Taken together, P2Y2 plays a critical role in regulating the pysiological function of fibroblasts of different tissues and mediating various pathological processes.

The current data showed that P2Y2 played an important role in fibroblast activation, ECM deposit, and muscle atrophy after denervation. Many other genes and / or pathways were involved in the pathophysiological changes of muscle injury and repair. Transcriptome analyses demonstrated that many physiological processes and genes were implicated in muscle atrophy and fibrosis [34–36]. A transcriptional signature of skeletal muscle atrophy was identified in human and mouse fasting muscle, which including 35 upregulated genes and 40 downregulated genes in both human and mouse skeletal muscle after fasting [34]. Moreover, 4 distinct transcriptional phases were identified in rat tibialis anterior muscle after denervation and genes involved in endocytosis, phagocytosis, extracellular matrix organization, and collagen fibril organization were enriched in phase III and IV, suggesting the activation of the biological processes of muscle atrophy and fibrosis [36]. An analysis of LncRNA found that phosphatidylinositol 3-kinase/Akt singaling pathway regulated genes laminin, collagen 5, and collagen 6 were activated in mouse muscle after contusion, indicating the overlapping of muscle regeneration and fibrosis after injury [37]. Deletion of cellular inhibitor of apoptosis 1 (cIAP1) or pharmacological inhibition of cIAP1 attenuated denervation caused muscle atrophy in mice [25]. Overexpression of TNF-like weak inducer of apoptosis (TWEAK) resulted in atrophy, fibrosis, fiber-type switching, and the degradation of muscle proteins after muscle denervation [38]. Ectonucleoside triphosphate diphosphohydrolases (ENTPDs) hydrolyzed extracellular ATP and inhbited the pro-fibrotic responses to ATP of cardiac fibroblasts [39], which implicated the roles of P2Y2 in nucleotide-induced fibrosis.

## Conclusions

The current study demonstrated that P2Y2 promoted the proliferation, migration, and activation of skeletal muscle fibroblasts and idenfied AKT, ERK, and PKC as its downstream signaling pathways. Knockout P2Y2 gene in mice alleviated denervation-induced muscle atrophy and fibrosis, which suggests that P2Y2 could be a novel target for treating muscle loss and fibrosis.

## Supplementary Information

**Additional file 1: Fig. S1.** Characterization of primary skeletal muscle fibroblasts. Fibroblasts isolated from mouse leg skeletal muscle were staining with antibodies against α-SMA (Fibroblast marker) and MyoD (myoblast marker) and analyzed with flow cytometry.

## Data Availability

The datasets used and/or analyzed during the current study are available from the corresponding author on reasonable request.

## Abbreviations

P2Y: P2Y purinoceptor
AKT: Protein kinase B
ERK: Extracellular-signal-regulated kinase
PKC: Protein kinase C
TGF-β1: Transforming growth factor β1
α-SMA: α-smooth muscle actin
CTGF: Connective tissue growth factor
MMP: Matrix metalloproteinase
ECM: Extracellular matrix
RT-qPCR: Quantitative real-time polymerase chain reaction
