# Supplementary material for: P2Y2 promotes fibroblasts activation and skeletal muscle fibrosis through AKT, ERK, and PKC
Source: BMC Musculoskelet Disord. 2021 Aug 11;22:680. doi: 10.1186/s12891-021-04569-y (PMC8359595; doi:10.1186/s12891-021-04569-y)

Fig. S1 Characterization of primary skeletal muscle fibroblasts. Fibroblasts isolated from mouse leg skeletal muscle were staining with antibodies against α-SMA (Fibroblast marker) and MyoD (myoblast marker) and analyzed with flow cytometry.

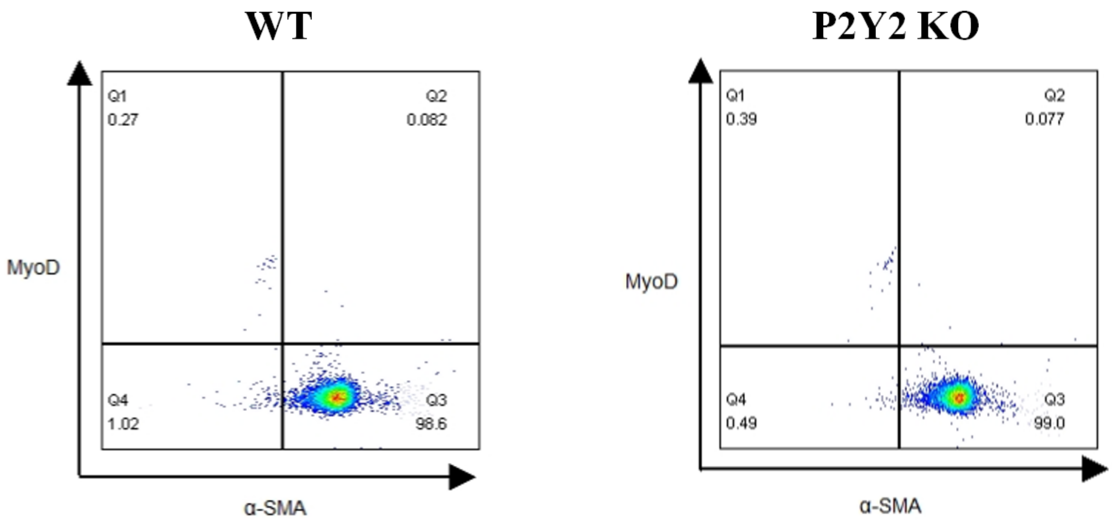

Supplement: Supplementary file 1 — Additional file 1: Fig. S1. Characterization of primary skeletal muscle fibroblasts. Fibroblasts isolated from mouse leg skeletal muscle were staining with antibodies against α-SMA (Fibroblast marker) and MyoD (myoblast marker) and analyzed with flow cytometry. [file 12891_2021_4569_MOESM1_ESM.pdf]
